# Understanding health systems challenges in providing Advanced HIV Disease (AHD) care in a hub and spoke model: a qualitative analysis to improve AHD care program in Malawi

**DOI:** 10.1186/s12913-024-10700-1

**Published:** 2024-02-26

**Authors:** Thulani Maphosa, Lise Denoeud-Ndam, Lester Kapanda, Sarah Khatib, Lloyd Chilikutali, Eddington Matiya, Boswell Munthali, Rosalia Dambe, Brown Chiwandira, Bilaal Wilson, Rose Nyirenda, Laywell Nyirenda, Bongani Chikwapulo, Owen Madeira Musopole, Appolinaire Tiam, Leila Katirayi

**Affiliations:** 1 Elizabeth Glaser Pediatric AIDS Foundation, Lilongwe, Malawi; 2 Elizabeth Glaser Pediatric AIDS Foundation, Geneva, Switzerland; 3 George Washington University, Washington, DC USA; 4 Department of HIV and AIDS, Ministry of Health, Lilongwe, Malawi; 5 Ministry of Health, Quality Management Directorate, Lilongwe, Malawi; 6 Elizabeth Glaser Pediatric AIDS Foundation, Washington, DC USA

**Keywords:** HIV, Advanced HIV, Health systems challenges, Hub and spoke model, Malawi

## Abstract

**Background:**

Despite tremendous progress in antiretroviral therapy (ART) and access to ART, many patients have advanced human immunodeficiency virus (HIV) disease (AHD). Patients on AHD, whether initiating ART or providing care after disengagement, have an increased risk of morbidity and mortality. The Elizabeth Glaser Pediatric AIDS Foundation (EGPAF) launched an enhanced care package using a hub-and-spoke model to optimize AHD care in Malawi. This model improves supply availability and appropriate linkage to care. We utilized a hub-and-spoke model to share health facility challenges and recommendations on the AHD package for screening and diagnosis, prophylaxis, treatment, and adherence support.

**Methods:**

This qualitative study assessed the facility-level experiences of healthcare workers (HCWs) and lay cadres (LCs) providing AHD services to patients through an intervention package. The study population included HCWs and LCs supporting HIV care at four intervention sites. Eligible study participants were recruited by trained Research Assistants with support from the health facility nurse to identify those most involved in supporting patients with AHD. A total of 32 in-depth interviews were conducted. Thematic content analysis identified recurrent themes and patterns across participants’ responses.

**Results:**

While HCWs and LCs stated that most medications are often available at both hub and spoke sites, they reported that there are sometimes limited supplies and equipment to run samples and tests necessary to provide AHD care. More than half of the HCWs stated that AHD training sufficiently prepared them to handle AHD patients at both the hub and spoke levels. HCWs and LCs reported weaknesses in the patient referral system within the hub-and-spoke model in providing a linkage of care to facilities, specifically improper referral documentation, incorrect labeling of samples, and inconsistent availability of transportation. While HCWs felt that AHD registers were time-consuming, they remained motivated as they thought they provided better patient services.

**Conclusions:**

These findings highlight the importance of offering comprehensive AHD services. The enhanced AHD program addressed weaknesses in service delivery through decentralization and provided services through a hub-and-spoke model, improved supply availability, and strengthened linkage to care. Additionally, addressing the recommendations of service providers and patients is essential to improve the health and survival of patients with AHD.

**Supplementary Information:**

The online version contains supplementary material available at 10.1186/s12913-024-10700-1.

## Background

Malawi has one of the highest HIV prevalence rates in the world, with an HIV prevalence of 8.9% among adults aged 15–49 years, corresponding to approximately 946,000 adults living with HIV, according to MPHIA 2020–2021 [1]. HIV and AIDS remain the most common causes of death in Malawi, with an estimated 13,000 deaths from AIDS in 2021 [1, 2]. Despite tremendous progress in improving antiretroviral therapy (ART) access through testing and treatment strategies in Malawi, a significant proportion of patients have advanced HIV disease (AHD), which has remained constant in recent years despite ongoing improvements in access to antiretroviral therapy (ART) [3]. AHD is defined by the World Health Organization (WHO) as a CD4 cell count < 200 cells/mm3 and/or stage III or IV disease in children older than 5 years and adults [4].

In Malawi, 28% of patients present with AHD at the time of the initial HIV diagnosis. Additionally, an increasing number of patients receive care at an advanced stage of HIV, following disengagement from treatment [5]. In Malawi, 22% of patients returned to AHD care. Patients with AHD are at a higher risk of death even after starting/restarting treatment, with the risk increasing with decreasing CD4 cell counts, especially with a CD4 cell count of less than 100 cells/mm^3^ [6]. The leading causes of mortality among adults with AHD include tuberculosis (TB), severe bacterial infections, cryptococcal meningitis (CM), toxoplasmosis, and *Pneumocystis jirovecii* pneumonia (PCP) [7–9].

The WHO has developed guidelines for managing AHD to reduce morbidity and mortality in patients with AHD. This package of interventions includes rapid ART initiation, enhanced screening and treatment, and/or prophylaxis for major opportunistic infections such as TB and CM and strengthened adherence support, including tailored counseling to ensure optimal adherence to the AHD care package [4].

The Elizabeth Glaser Pediatric AIDS Foundation (EGPAF) supported an AHD intervention model to optimize the services offered to patients with AHD in Malawi. The integrated AHD package is built on WHO guidelines and includes enhanced decentralization of care through a hub-and-spoke model to bring AHD care geographically closer to patients. An enhanced package of AHD interventions was implemented, including decentralized CD4 tests, TB-lipoarabinomannan antigen assay (LAM), and initiation of prophylaxis, including TB preventive therapy (TPT) and cotrimoxazole prophylaxis (CPT) [4]. The facilities adopted a model for improvement used in quality improvement approaches to respond to system issues that fall within their control. A prioritization matrix was used, which focused on addressing problems that required fewer resources, less time to respond, and a higher impact on AHD care. Locally formulated solutions complemented the system improvements made at the national level. The team met weekly to review their response plans, termed the Plan Do Study Act (PDSA), to address weak client flow, stock management inefficiencies, and human-related bottlenecks. Quality improvement (QI) teams have innovated around AHD care bottlenecks to drive the uptake of newly introduced treatment regimens for opportunistic infections and strengthen their pharmacovigilance.

A hub-and-spoke model was used to deliver appropriate healthcare services to the peripheral sites. A hub site was defined as a health facility with comprehensive packages of AHD care that managed patients requiring hospitalization. Spoke sites offer outpatient and routine care to stable AHD patients, and refer critically ill patients to nearby hubs. This hub-and-spoke model was implemented to create linkages between referral networks and provide strengthened community-based care to reduce the risk of loss to follow-up. As part of the evaluation, the project conducted a qualitative assessment to examine the views of healthcare service providers on AHD care. This study focused on the population of healthcare providers, but it is important to note that as part of this qualitative evaluation, we also conducted in-depth interviews (IDIs) with HIV-positive men and women aged 18 years or older who received AHD services for at least two months in the same facilities. The results from these patients were shared in another study.

### Objectives

This study assessed providers’ experiences of implementing an enhanced package of AHD care through the hub-and-spoke model, aiming to contribute to a more comprehensive understanding of the challenges in delivering optimal care to AHD patients. Based on their suggestions, we identified ways to strengthen service delivery for patients with AHD.

## Methods

### Study design

This qualitative study used in-depth interviews (IDIs) with healthcare workers (HCWs) and lay cadres (LCs) to explore facility-level experiences in providing an enhanced package of AHD services.

### Site selection

Four sites were purposively selected from the intervention sites, including two hubs and two spokes. Hub sites are larger sites that supports smaller ‘spoke’ sites by providing supplies, technical support and accepting patients with more complicated health problems. Spoke sites provide services at the community level, and offer basic care. These sites are located in the Dedza and Ntcheu districts in the Central Region. Mchinji and Ntcheu District Hospitals are public hub sites dedicated to providing comprehensive secondary-level care under the MoH while Sister Theresa Hospital is a private hub site in the Ntcheu district that operates under the Christian Health Association of Malawi (CHAM). Of spoke the sites, Mtemdere Health Center is a private health facility in Dedza under CHAM, which delivers primary care within a rural setting while, Lizulu Health Center is also a is a public health facility in Ntcheu under MoH in an urban setting.

The selection was based on the area with the highest patient volume. The sites were selected to have equal numbers of rural and urban locations.

### Study population

The study population included those involved in delivering HIV care at four selected sites, including HCWs and LCs. Eligible HCWs were involved in HIV care at the intervention sites, including laboratory staff, ART nurses, and clinically trained staff including clinical officers (COs), medical assistants (MA), and medical doctors (MDs) who consented to participate. HCWs in training were excluded from the study.

Due to the shortage of clinically trained medical professionals in Africa, ‘lay cadres,’ those not clinically trained, often provide critical support, including counseling, follow-up, tracking, etc. to health care patients [10]. Lay workers involved in the screening and follow-up of PLHIV were eligible for LCs. This group included expert clients (ECs), Health Surveillance Assistants (HSAs), HIV Diagnostic Assistants (HDAs), ART clerks, adherence support officers (ASO), and hospital attendants. ECs that were not involved in the screening and follow-up of patients were excluded.

### Participant recruitment

Convenience sampling was used to recruit participants. To recruit HCWs, research assistants (RAs) spoke with the health facility nurse to identify eligible participants who provided AHD services to patients and were present on days when data collection occurred at the facility. There was more emphasis on recruiting ECs because they are generally more involved in the counseling and follow-up of patients at their homes. In the selected health facilities, all HCWs and LCs that met the inclusion criteria were included in the study.

### Data collection

The RAs were previously experienced in collecting data and participated in 40-hour data collection training to familiarize themselves with the study protocol, data collection tools, human subjects, and the standard operating procedures (SOPs) of the study. The training involved role-playing and examinations to ensure that the RAs fully grasped the materials.

The data were collected between December 2021 and February 2022. Written informed consent was obtained from all study participants prior to data collection. All the data were collected from a health facility.

The IDIs were conducted with study participants using semi-structured IDI guides (Supplementary File 1, Supplementary File 2, Supplementary File 3). The guides included topics such as knowledge required for the diagnosis and treatment of patients with AHD, perceptions of one’s own self-efficacy in diagnosing and treating patients presenting with AHD, experiences providing AHD services, and how the enhanced AHD program affected job satisfaction (Supplementary File 1, Supplementary File 2, Supplementary File 3). IDIs were led by RAs who were hired and trained by the EGPAF. The RAs used semi-structured interview guides and probes to obtain in-depth responses during the interview process. All IDIs were audio-recorded and completed in English or Chichewa depending on the participant’s preference. The Interviews lasted–50–90 min. The recorded IDIs were translated from Chichewa into English as appropriate. IDIs were transcribed by RAs soon after data collection to minimize recall errors. The interview transcripts also documented descriptions of the participants’ nonverbal responses, referred to as ‘field notes. The participants’ demographic data were also collected.

Thirty-two IDIs were conducted, including 16 for HCWs and 16 for LCs. Previous literature determined that saturation was reached in 12 interviews [11].

### Analysis

The data were analyzed using a thematic analysis. A short-answer analysis was conducted, which consisted of two research assistants manually compiling all the responses per question from the interview transcripts. The collected responses were then analyzed for overarching themes derived from the data, and the findings were summarized using text-based summaries and data display tables. In the data display tables, data were separated by study population and study site type (hub vs. spoke), and compared for similarities and differences. Textual data were carefully analyzed to identify recurrent patterns and themes. Text excerpts representing identified themes were included.

## Results

The mean ages of HCWs and LCs were higher at spoke facilities at 37 years of age than at hub facilities at 35 years of age. There were three clinicians, one registered nurse, two laboratory technicians, and one nurse midwife from the hub site. From spoke sites, there were three clinicians, four nurse midwife technicians, one adherence and support officer (ASO), and one hospital attendant. Notably, more female HCWs and LCs were present at spokes than at hubs. Hub facilities also had more HCWs and LCs working for more than ten years in their positions, with 20% working for more than ten years at hubs, compared to 13% working for more than ten years at spokes (Table 1).

**Table 1:** Demographic data for healthcare workers and lay cadres at hub sites and spoke sites

	Spoke (*N* = 16) N (%)	Hub (*N* = 16) N (%)
Age (years)		
**Median age [IQR]**	37.5 [7–53]	35.13 [9–35]
Gender		
**Male**	5 (31)	8(50)
**Female**	11(69)	8(50)
Position Type		
**Clinician (medical assistant /clinical officer / medical doctor)**	3(19)	3(19)
**Reg nurse**		1(6.3)
**Lab technician**		2(13)
**Nurse Midwife technician**	4(25)	1(6.3)
**Nurse tech**	1(6.3)	
**Registered Nurse Midwife**		1(6.3)
**Adherence Support Officer**	1(6.3)	2(13)
**Hospital Attendant**		1(6.3)
**Expert Client**	4(25)	2(13)
**HIV Diagnostic Assistant**		2(13)
**ART Clerk**	3(19)	1(6.3)
Duration worked in position (years)		
**< 1**	1(6.3)	
**1–5**	9(56)	10(67)
**6–10**	4(25)	2(13)
**> 10**	2(13)	3(20)

### Themes

The results were organized into four themes: perceived benefits of improved AHD packages of services, positive changes reported with the new AHD program, challenges in providing AHD services to patients and solutions, and HCWs and LC recommendations.

### Perceived benefits of new AHD care package according to healthcare workers and lay cadres

The participants highlighted that introducing quality improvement (QI) sessions under AHD care added value to their work, because they could discuss patient welfare and make appropriate decisions. They felt that QI brought additional value to their services and facilities because they could see their work’s direct impact on improving patient care in real time. HCWs at spoke sites said that they felt better equipped to handle AHD patients and provide better services by identifying gaps and conditions they may have previously missed (Table 2). They felt that this resulted in the AHD program helping with timely treatment initiation (Table 2).

**Table 2:** Benefits of the improved AHD package of services

Theme	Benefits of the improved AHD package of services
(1) Benefits of the quality improvement system	*It [quality improvement] has added a certain kind of value to our work because when the results are shared in real time, it means that the patient will receive the necessary treatment in time, which is quite different from the previous days where when you collect a sample from an AHD patient, we were telling them to wait at home for one week before they return to the facility. However, this new process is helping our clients to start treatment on time.* (Male HCW, Hub, Lab technician, 50) *We can track the progress of the work we are doing here and be able to recognize our flaws and be able to rectify the problem* (Female HCW, Spoke, Nurse midwife technician, 27) *Quality improvement has helped us so much that we can sit down and discuss our problems based on the reports. We take the issues to the QI and then discuss how we can improve the problem that we have as a hospital. For example, a separate clinic was specially made for AHD patients because it was realized that they were not receiving the necessary assistance required when information was given to them as a whole group. That decision was made based on Quality improvement.* (Male LC, Hub, Adherence Support Officer, 28)
(2) Improved job satisfaction	*This has just motivated us to have a strong desire to stay focused on our clients and see that everyone is getting the proper support* (Female LC, Spoke, ART Clerk, 46) *It [AHD program] has improved my satisfaction because I can see the change in the work we do; I can screen assess in any way possible, and not only are we targeting clients who have AHD but also the new ones so that we can be able to classify them* (Male HCW, Hub, Nurse Midwife Technician, 27) *I am satisfied when I see the excellent results that are coming out because I believe that with the coming of the AHD as a program, the main goal is that we should reduce the mortality rate for those patients that are living with HIV so because we are following this procedure, we are reducing ART deaths. Secondly, it is also encouraging us that most patients are now having their viral load suppressed since we do not want to have more patients having a high viral load, which will show that we need to work harder. So when we noticed that we had an AHD patient, and later on, because of this AHD program when the person’s sample was collected and tested for viral load. The results are suppressed viral load such that it is undetectable; we know that we are working tirelessly for this, and we are happy to see such results.* (Male HCW, Hub, Reg N/M, 47)
(3) Increased ability to care for AHD patients	*Our work in this program has improved the care that AHD patients get from this facility. We were missing some things in the past because of a lack of knowledge, and we lost some of our patients. However, with the coming of AHD as a program, we can know that when our HIV patient is admitted to the ward, we need to screen them for other infections. In the screening process, we diagnose some other diseases like cryptococcal meningitis, and as soon as we analyze them, we prescribe drugs to this patient. At the end of everything, we can save a life, unlike when our patients were admitted to the wards without us knowing what was happening to them. So, the coming of AHD as a program has improved the care that AHD patients receive from this facility. We know now that this patient has such conditions, and this one has this condition.* (Female HCW, Spoke, Nurse midwife tech, 30)

HCWs and LCs in both hubs and spokes reported that improvements in patient care through AHD-related services resulted in increased job satisfaction. HCWs described feeling empowered because they saw progress in their patients’ health, fewer deaths and better health outcomes (Table 2). Overall, they feel motivated and have a strong desire to stay focused on the client’s welfare, and see that everyone receives proper support (Table 2).

HCWs and LCs also described feeling more empowered and equipped to provide AHD care for their patients. LCs reported that they were now able to provide more support to defaulters as opposed to just giving patients their medications and sending them home (Table 2).

At both spokes and hubs, the HCWs expressed that they could directly observe the impact of their work on improving patient care. HCWs at spoke sites said that they feel better equipped to handle AHD patients and provide better services by identifying gaps and conditions they may have previously missed (Table 2).

### Positive changes from the AHD program

HCWs and LCs across hub and spoke sites discussed enhanced AHD services and how they strengthened their ability to treat patients. Most HCWs said that they are now more capable and confident of screening for opportunistic infections among patients with AHD. Many said they had testing and treatment available at their facilities. Because of the improved AHD services available owing to the program, HCWs mentioned that they engaged with patients more and could provide tailored care depending on their needs (Table 3).

**Table 3:** Positive changes from the AHD program

Theme	Positive change due to the AHD program
Availability of additional AHD services	*We can now provide our patients with better services; we can track everything and not miss any condition in a patient.* (Female HCW, Spoke, Clinician (MA/CO/MD, 28) *So, AHD as a program is good because patients can get assistance not only for HIV but also for other conditions which, at first, they were ignoring but with the coming of this AHD program, everyone is screened to discover if s/he is developing some conditions that can be treated before they reach the climax.* (Female LC, Spoke, ART Clerk, 41). *Yes, in the past, we would spend less time with our patients because we were not doing all the screening that we are doing now, but now all the patients are being screened for other diseases once found HIV positive.* (Female HCW, Spoke, Nurse Midwife Tech, 40).
Increased training to identify AHD patients and for follow-up	*We have the ability. Firstly, we use all the methods that we learned from the training, such that when a person defaulted from taking their drugs when we follow up on him, or she and s/he have returned to care, especially if s/he is at an advanced stage of HIV, and s/he has gone beyond WHO stages precisely when s/he is on either stage 3 or stage 4. We have the ability because when we are chatting with the patient, we notice such conditions that this patient has reached the advanced stage of HIV disease. So, after that, there is that chance that we test for CD4 count and then go further to other tests.* (Male HCW, Spoke, Nurse Tech, 46) *The training opened up some important topics we previously did not know, but since we attended the training, we have been able to help out AHD patients by referring them to the right doctor for the proper treatment*. (Female LC, Hub, HDA, 27).
Increased mentoring and support in providing AHD services	*We have mentors who train us to care for people and our friends from EGPAF; sometimes, they orient us and have collaborative meetings. Friendly, we learn what our friends are doing so that we can assist AHD patients.* (Female HCW, Hub, Reg nurse, 32). *Yes, we received the mentorship, and when we experience problems, we can ask to say we do not understand here; how will we do this? Moreover, sometimes, you need help understanding the screening process, and we still ask them.* (Female HCW, Spoke, Nurse Midwife Tech, 30).

Furthermore, HCWs and LCs have also reported an increased ability to identify patients with AHD due to training. They specifically mentioned that they could better identify patients who required special care and were better equipped to follow-up with them (Table 3).

HCWs noted increased mentoring and support in providing AHD services. HCWs expressed that they were satisfied with the mentoring necessary to provide care to patients with AHD. They also explained that they could seek guidance and support when they experienced difficulties in providing care to patients with AHD (Table 3).

**Service providers need help in providing AHD services.**

Half of the healthcare workers felt that AHD training required improvement. The main concerns were the short duration of training, insufficient information provided during training, and challenges with frequent staff turnover requiring the training of new staff. HCWs at spoke sites said that training sessions needed to be longer. HCWs at spoke sites also complained that additional training was required because of heavy staff turnover. LCs from both hubs and spokes reported that they found it challenging to learn secondhand skills from others who had been trained and were now training them. LCs at hub sites further explained that the training they received from their peers needed to be improved and that they needed more information (Table 4).

**Table 4:** Challenges in providing AHD services to patients

	Challenges in providing AHD services to patients
Health Care Worker Training	*I am the only one who attended the orientation [training] out of 3 clerks, which means that those who did not participate in the orientation do not know more about it apart from the information that those who attended the orientation, like me, shared with them.* (Female LC, Spoke, ART Clerk, 41) *Our friends [co-workers] advised us [information based on the training they attended]. However, second-hand information is complex because the way they understand the information in class and what they can grasp is different. I believe they did not explain everything, and some things were other because there is an element that while they were learning, they might have been on their phone, or they were thinking about something else, or they were outside, so they may not have explained everything because they missed it.* (Female LC, Hub, Adherence Support Officer, 31)
Supply availability	*We often refer to significant hospitals because we need the necessary supplies to conduct the tests.* (Female HCW, Spoke, Clinician (MA/CO/MD), 29). *We need cartridges to conduct a CD4 cell count, but the supplier still needs to give us those things, so we cannot do a CD4 Cell count.* (Male HCW, Hub, Nurse Midwife tech, 27).
Patient referral forms	*The main problem could be poor communication; the patients are being referred here [from the hubs] but need more documentation.* (Female HCW, Spoke, Nurse Midwife tech, 29) *Some challenges include labeling the samples; the labeling is done differently from the one on the form… most of the columns are not filled. For example, like staging, they do not fill anything on that. So, when they do not feel on the stage of the patient’s sample, it becomes difficult for us to conduct such test on such sample here at our laboratory because we do not know what difficulties we are supposed to conduct since staging guides us to know that for this sample, we are supposed to conduct such test. We have been trying to communicate with them about it, but they do not change; maybe they need another orientation on how to fill in the form.* (Male HCW, Hub, Lab technician, 50) *Patient referral systems: The issue is the means of transport. Suppose the means of transportation is available, and the ambulance has enough fuel. In that case, it is not that difficult to transfer an AHD patient from a spoke site to here as a hub site or transfer that same AHD patient from here [hub site] to a spoke site that is within the catchment area of their village where they will continue getting their medication. It is easy if the vehicle is available or if fuel is enough to cater for the transfer* (Female HCW, 27).
Increased workload	*Healthcare worker challenges providing services*: *The AHD program increased our workload, which is vital because we assist patients fully, and it is an added task for one to have fully helped a patient without missing any condition.* (Female HCW, Spoke, Clinician (MA/CO/MD), 28).

While HCWs and LCs in both hubs and spokes described medications as almost always available to patients, many HCWs and LCs expressed concern about their facilities’ lack of available supplies. They noted the need for more sample collection kits for CD4 tests and cartridges for the TB LAM tests. HCWs and LCs at both hub and spoke sites described inadequacies in the equipment and machines used to run tests, including point-of-care (PoC) machines, CD4 counters, and chemical analyzers. Both site levels also reported that only a few staff members knew how to use the machines. Some HCWs and LCs at spoke sites reported staff shortages when collecting samples for PoC machines. HCWs and LCs at hub sites noted that the additional machines lacking in the facilities were chest X-ray machines, abdominal ultrasound scanners, and GeneXpert machines. HCWs stated that when spoke sites ran out of supply, they requested kits from the hub sites. Therefore, hub sites were overwhelmed by receiving requests from the two spoke sites (Table 4).

HCWs from hubs said that the referral system to transfer AHD patients was going well because they had ambulances that could transport the patients, whereas most HCWs from spoke sites noted that they faced issues with transportation transfers. One of the main challenges reported by HCWs at spoke sites is that the ambulance system must be fixed to transport AHD patients to hubs. They said that ambulances were not always available, or sometimes did not have a petrol. This would leave patients to find their mode of transportation, which could be challenging and unsustainable owing to financial challenges. HCWs at both hubs and spokes said that they faced issues and difficulties with the vehicle when referring to patients with AHD. HCWs at spoke sites reported problems with referral documentation for patients arriving at the facility, including the need for proper health passports, written treatment history information, and referral letters. Many HCWs at spoke sites explained that the biggest challenge in transferring patients is their reluctance to transfer care, because they prefer to go to the same facility with which they are familiar and comfortable (Table 4).

HCWs at hubs have noted that delays in receiving laboratory results create challenges in diagnosing patients with TB. In addition, hub site HCWs reported that spoke sites must correctly fill out the information for the samples. HCWs at hubs and spokes said that the space to enter the text in the register was too small, and they found it challenging to fill it out. Furthermore, transportation for home visits was noted as a challenge for providing AHD services. HCWs noted that adding clients who were not from Malawi to the register and following up with them was difficult. HCWs at spokes reported challenges dealing with patients who were in denial of their diagnosis and unprepared to begin treatment (Table 4).

HCWs at hubs and spokes reported that new AHD training and service provision increased their workload. The increased workload is attributed to more rigor in assisting patients adequately without missing any conditions, which are considered additional tasks. They noted that completing the new protocol and the AHD register was time consuming. However, they remain motivated and know that they provide improved patient care and services. (Table 4).

### HCW and LC requests & recommendations

HCWs reported that a constant availability of supplies and equipment is needed to improve their ability to provide care for patients with AHD. They requested that equipment, including CD4 count machines, X-ray machines, viral load machines, and FBC machines be made available. They highlighted the need for an adequate supply of CD4 cartridges and sample collectors for serum Cr-Ag and urine LF-LAM testing. HCWs also noted that healthcare worker capacity must be increased to provide better AHD care, including hiring additional staff members such as lab technicians and clinicians. HCWs recommended establishing meetings with HCWs from hub and spoke sites to discuss gaps in AHD treatment guidelines (Table 5).

**Table 5:** Recommendations from HCWs and LCs to improve AHD care across hubs and spokes

Theme	Recommendations from HCWs and LCs
Supplies and equipment	*We need enough equipment always available for AHD; yes, they say cartilages for CD4 are expensive, but we need them, supply for material for screening should also be available, and if or when we run out of these things, they should quickly resupply.* (Female HCW, Spoke, Clinician (MA/CO/MD), 29)
Training	*Providing additional training and conducting refresher training to those healthcare workers that were trained way back would help improve our ability to provide care to AHD patients.* (Female LC, Hub, HIV Diagnostic Assistant, 27) *On the diagnosis, it [training] was sufficient, but on the management, it [training] needed to be increased, and we need the necessary medication at this facility. On top of that, our health workers needed to be trained on how this new treatment. So, if one client is diagnosed with cryptococcal meningitis, they are sent to the District Hospital on specified dates.* (Male HCW, Hub, Nurse Midwife Tech, 27)
Counseling	*Because the person has a low CD 4 count, without counseling, it does not work [adherence], but if they can train and explain to us [lay counselors] properly, we would be the ones doing the counseling.* (Female LC, Hub, Hospital Attendant, 49) *It usually involves telling the patient the ramifications of skipping taking medicine and the benefits of taking medication faithfully; it also involves letting them understand that they need to set their own time for taking the medication without being pressured to follow a particular schedule. We also help counsel our very own AHD patients.* (Female HCW, Hub, Clinician (MA/CO/MD), 24).
Mentoring/supervision	*[Another main request was] knowledge, continuous mentorship, and supervision. Those who do not know should know; on care, they should monitor our data.* (Male HCW, Hub, Lab technician, 35)
Home visits	*We need to strengthen our relationship with our AHD clients by at least visiting them [at home] twice a month so that we can know how they are doing or how things are going out for him or* them. (Female LC, Spoke, Expert client, 45).

HCWs at both hub and spoke sites emphasized the need for additional training on many topics. Hub sites reported that spoke sites required more training on labeling samples because they arrived incorrectly labeled, causing challenges in determining which tests to conduct at the hub sites. This prevented them from running the tests because they were unaware of the reasons for testing. HCWs said that they needed training in CM, including questions regarding drug preparation, administration, and different treatment plans for CM. Furthermore, training was requested on new topics including viral load interpretation and liver function tests. HCWs also requested training on best practices to keep records organized in the registry (Table 5).

HCWs emphasized the need for additional training on counseling skills to help them better advise patients and strengthen patient adherence to care. This would also help them address the challenges faced by the defaulters. Finally, the HCWs stated that they wanted refresher training to keep their knowledge updated and for continuous mentorship and supervision (Table 5).

HCWs noted that additional support was needed to strengthen adherence through community-based support/home visits. LCs from both hubs and spokes mentioned the need for more help in home visits, including other allowances, to increase the frequency of visits. They also wanted food parcels to provide clients with when they visit their homes. They described difficulties during the rainy season and requested raincoats and transportation support for home visits (Table 5).

### Implementation of real-time mitigation efforts

This study was conducted as operations research to identify the challenges of the enhanced AHD program and implement solutions to the identified problems in real time. The solutions were implemented after data collection; therefore, interview data were unaffected. It is essential to acknowledge the steps that are required to address these challenges.

To address the inadequacies in training, facility orientations are now jointly provided by deployed AHD clinicians and trained facility Ministry of Health staff. Regarding the increased workload resulting from the enhanced package of services, facilities have used data review meetings at the ward level to motivate a team with progress made in patients’ health. Concerns about increased workload dissipating over time. Finally, regarding the request for increased mentorship and supervision, the QI team successfully lobbied for the retention of specific leaders of the AHD program in the wards and facilities until the upcoming team was sufficiently acquainted with the processes and delivery of the AHD services.

HCWs and LCs have described solutions they have implemented to help address the gaps in delivering AHD care to patients. HCWs at both hubs and spokes reached out to head staff members or district health officers when they were low in supply and equipment. Regarding staff shortages, HCWs at hub sites said that they had learned the tasks of other positions to provide support when needed. HCWs also provided additional counseling to patients who were not ready to start treatment. For patients who lived far away, HCWs said that they would occasionally send medications to other community members. Lastly, HCWs at spokes reported using WhatsApp to send test results and avoid further delays and noted that bike and scratch cards helped them reach patients needing AHD care. If a patient was too ill, LCs reported that they could counsel a guardian or wait until the patient was sufficiently stable to counsel. For patients who moved out of Malawi, LCs at spokes attempted to contact them through relatives in the village; if unsuccessful, they were documented in the register that the patient had left.

## Discussion

Our study confirms the successful implementation and acceptance of an enhanced WHO-recommended package for Advanced HIV Disease (AHD) care echoes and consolidates the existing body of literature. Numerous studies support our findings. Frank et al. [12] and Gupta et al. [13] echoed similar success stories in different regions, highlighting the consistent feasibility and acceptability of enhanced care packages in diverse healthcare settings. Additionally, studies conducted by Thurman et al. [14] and Musengimana et al. [15] not only reiterated the feasibility but also emphasized the significant impact of these packages in improving patient outcomes, reinforcing the importance of their implementation in combating the AIDS epidemic.

HCWs highlighted the crucial challenges inherent in the patient referral system, emphasizing the paramount need for comprehensive paperwork and documentation to ensure a seamless connection of care between central hubs and peripheral facilities. This finding echoes the findings of various studies that underscore the pivotal role of well-functioning referral systems in initiating care and mitigating patient disengagement. For example, research conducted in Tanzania not only emphasized the significance of providing referral forms but also demonstrated how these forms facilitated swift patient entry into healthcare facilities or directed them to appropriate clinics, significantly streamlining the care continuum [16]. Similarly, investigations in Mozambique corroborated these findings by stressing the indispensable nature of referral slips, showcasing their efficacy in expediting treatment at health facilities and ensuring the unbroken continuity of care [17]. Furthermore, the challenges posed by transportation issues within the patient referral system have been reiterated in the literature, emphasizing the critical role of proximity and access to transportation in optimizing the linkage of care [18]. Studies have consistently highlighted that accessibility and availability of transportation resources significantly impact the efficiency of care linkages, thereby emphasizing the necessity for viable transport options to ensure timely and continuous healthcare access for patients within these referral systems [19].

HCWs underscored critical concerns regarding the scarcity of supplies and equipment within healthcare facilities, highlighting the shortage of test kits as a significant impediment to the delivery of adequate care. Insufficient supply, notably in point-of-care (POC) CD4 testing, creates substantial challenges and frustration for HCWs involved in providing care for Advanced HIV Disease (AHD). Disturbances in equipment and shortages significantly contribute to diagnostic hurdles and delayed test results. When spoke sites exhaust their supplies and rely on central hub sites, they strain the latter’s resources, causing disruptions in service provision and amplifying the burden on these hubs [18]. Effective stock management has emerged as a critical determinant in addressing these issues, with research demonstrating that well-organized inventory control significantly mitigates delays in turnaround times and ensures the availability of essential supplies for uninterrupted care delivery. Additionally, studies have emphasized the importance of innovative stock management strategies, such as predictive modeling and real-time inventory tracking, in averting supply shortages and streamlining the distribution of resources across healthcare facilities, thereby enhancing the overall efficiency of care provision [20].

Addressing these inadequacies requires a strategic approach, including targeted training for HCWs that leverages pre- and post-test evaluations to identify and address skill gaps, consequently enhancing diagnostic and management proficiency. Research corroborates the significance of such targeted training programs, showing their effectiveness in improving HCWs’ skills and knowledge, thereby optimizing patient care outcomes [21]. Specifically, in the context of point-of-care (POC) testing, various studies have emphasized the criticality of comprehensive training modules that emphasize quality assurance, standardized documentation practices, proficiency testing, and thorough verification of new kit lots [22]. Structured training programs to boost HCWs’ confidence and competence in POC testing will ultimately ensure the accuracy and reliability of test results. Furthermore, innovative communication methods play a pivotal role in mitigating challenges associated with delays in test results and patient tracing. Studies have shown that employing instant messaging platforms such as WhatsApp or utilizing text messaging and telephone consultations facilitates prompt communication, aiding result dissemination and patient follow-up [23]. These findings underscore the efficacy of leveraging technology-enabled communication tools to streamline healthcare processes and improve patient engagement, ultimately contributing to more efficient and effective healthcare delivery.

Furthermore, HCWs emphasized the crucial necessity for home visit support, particularly for critically ill patients, citing challenges such as geographical distance, challenging terrain, and adverse weather conditions that significantly hinder timely and high-quality home visits. These obstacles have been consistently highlighted across various studies, highlighting the universal challenges faced by HCWs in reaching and providing care to patients residing in remote or geographically inaccessible areas. For instance, research conducted in diverse settings has reiterated the impact of geographical barriers, emphasizing the difficulties faced by HCWs in delivering timely and consistent home-based care to patients in remote or rural regions [24–26]. Moreover, adverse weather conditions exacerbate these challenges, disrupt healthcare access, and necessitate implementation of tailored strategies. Studies exploring healthcare delivery during extreme weather events or in geographically challenging areas have proposed innovative solutions, such as the utilization of specialized transport systems, community health worker networks, and telemedicine initiatives to ensure uninterrupted care delivery, especially during inclement weather or in geographically isolated regions [27, 28]. These findings underscore the need for adaptable and context-specific strategies to address geographical and weather-related challenges and to ensure continuous and high-quality care provision for the most vulnerable patient populations.

### Strengths and limitations

The main strength of this study lies in its use of a qualitative approach, which helped obtain a rich, complete, and in-depth exploration of the issues surrounding the feasibility and acceptability of an enhanced package of AHD care from diverse groups of service providers. The limitations include the subjective nature of the interviewees’ responses and the risk of social desirability to please the research team. Conducting interviews in the location of employment may have also contributed to social bias. In addition, the data were collected from only four sites, and HCWs and LCs at other sites may have faced different challenges. The study did not interview any former HCWs who may have left their positions; this group may have shared a different perspective.

## Conclusion

In conclusion, our study underscores the feasibility and acceptance of enhanced AHD care while pinpointing critical issues within patient referral systems, supply shortages, POC testing challenges, and the need for improved home visit support. Addressing these challenges requires a multifaceted approach, including streamlined referral systems, effective stock management, comprehensive HCW training, and innovative patient contact methods, to ensure sustained and optimal care delivery, particularly for vulnerable individuals. These findings suggest the importance of addressing weaknesses in the delivery of services within the hub-and-spoke model to comprehensively tackle facility-level challenges and ensure that they do not impede the services provided to patients with AHD. Adequately addressing the weaknesses in delivering services within the hub-and-spoke model, improving supply availability, and appropriate linkage to care is necessary to implement enhanced AHD care successfully.

### Electronic supplementary material

Below is the link to the electronic supplementary material.

Supplementary Material 1

Supplementary Material 2

Supplementary Material 3

Supplementary Material 4

## Data Availability

Data are available upon reasonable request to the corresponding author.

## Abbreviations

AHD: advanced HIV disease
ART: antiretroviral treatment
ASO: Adherence Support Officers
CO: clinical officer
EGPAF: Elizabeth Glaser Pediatric AIDS Foundation
EC: expert client
HCW: health care worker
HDA: HIV diagnostic assistant
HSA: Health Surveillance Assistant
IDI: in-depth interview
IRB: institutional review board
LC: lay counselor
MA: Medical Assistant
MD: medical doctor
RA: research assistant
TB: tuberculosis
WHO: World Health Organization
