# Supplementary material for: Understanding health systems challenges in providing Advanced HIV Disease (AHD) care in a hub and spoke model: a qualitative analysis to improve AHD care program in Malawi
Source: BMC Health Serv Res. 2024 Feb 26;24:244. doi: 10.1186/s12913-024-10700-1 (PMC10897989; doi:10.1186/s12913-024-10700-1)
Supplement: Supplementary file 1 — Supplementary Material 1 [file 12913_2024_10700_MOESM1_ESM.docx]

COREQ CHECKLIST- completed for **Understanding health systems challenges in providing Advanced HIV Disease (AHD) care in a hub and spoke model: A qualitative analysis to improve AHD care program in Malawi** manuscript.

November 27^th^ 2023

No Item Guide questions/description

Domain 1: Research team and reflexivity

***Personal Characteristics***

1. Which author/s conducted the interview or focus group?

*The interviews were conducted by research assistants (RAs) trained by EGPAF, please see the data collection paragraph within the methods section.*

2. Credentials- What were the researcher's credentials? E.g. PhD, MD

*Four RAs had a Bachelor of Science degree in Nursing and Midwifery and one lady had a Bachelor of Science in Public Health but also had a Diploma in Clinical Medicine.*

3. Occupation - What was their occupation at the time of the study?

Four RAs had worked as Nurses in NGOs and the other RA had worked as a Clinical Officer in NGOs. They all had more than 4 years of research experience -both Qualitative and Quantitative methods. In the data collection section of the methods part of the manuscript, we mention that the RAs had previous experience in data collection in the first paragraph of data collection, in the methods section.

4. Gender Was the researcher male or female?

*There were three female RAs and two Male RAs.*

5. Experience and training- What experience or training did the researcher have?

*The Research Assistants were previously experienced in collecting data and participated in a 40 hour data collection training to become familiar with the study protocol, data collection tools, human subjects and the standard operating procedures (SOPs) of the study. The training involved role-playing and exams, to ensure that the RAs fully grasped the material. This information has been added to the beginning of the data collection paragraph, in the methods section.*

***Relationship with participants***

6. Relationship established -Was a relationship established prior to study commencement?

None of the RAs new the sites prior to being engaged for the data collection activity. The RAs had no previous interaction with the Health care workers before data collection.

7. Participant knowledge of the interviewer -What did the participants know about the researcher? e.g. personal goals, reasons for doing the research

*During the informed consent process, participants learned the objective of the research and the role of the research assistant.*

8. Interviewer characteristics -What characteristics were reported about the interviewer/facilitator? e.g. Bias, assumptions, reasons and interests in the research topic

*The data collected focused on evaluating the new advanced HIV disease (AHD) care program at the health facility, and that questions were about program and its implementation. Due to the fact that the data collected did solicit information about the participant’s personal life or situation, interviewer characteristics were not included in the data collection description.*

**Domain 2: study design**

***Theoretical framework***

9. Methodological orientation and Theory What methodological orientation was stated to underpin the study? e.g. grounded theory, discourse analysis, ethnography, phenomenology, content analysis.

*It is stated in the analysis paragraph of the data collection section that thematic analysis was used to analyze the data.*

***Participant selection***

10. Sampling -How were participants selected? e.g. purposive, convenience, consecutive, snowball

*In the ‘participant recruitment’ paragraph of the methods section we have stated that convenience sampling was used to recruit participants.*

11. Method of approach -How were participants approached? e.g. face-to-face, telephone, mail, email.

*In the participant recruitment paragraph of the methods section, recruitment methods are stated.*

*‘To recruit HCWs, the research assistants (RAs) spoke with the health facility's nurse to identify eligible participants who provided AHD services to patients and were present on days that data collection occurred at the facility.’*

12. Sample size -How many participants were in the study?

*At the end of the data collection paragraph, within the methods section, we state the sample size.*

13. Non-participation -How many people refused to participate or dropped out? Reasons?

*No participants dropped out or refused to participate in the study*.

***Setting***

14. Setting of data collection -Where was the data collected? e.g. home, clinic, workplace

*It is stated in the first part of the data collection paragraph, within the methods section, that all data collection occurred at the health facility.*

15. Presence of non-participants -Was anyone else present besides the participants and researchers?

*No, the research assistants interviewed participants alone (standard practice).*

16. Description of sample -What are the important characteristics of the sample? e.g. demographic data, date

*The sample is described in the first paragraph of the results section. The sample size is rather small, so it’s difficult to make many claims about the demographic data. However, we did find it interesting that at the Hub sites, the gender of the population (16 people) was split equally between men and women, as usually the majority of HCWs are often female. We also thought the reader may find it interesting to see all of the different lay cadres involved in health care.*

***Data collection***

17. Interview guide *(See Attached Supplementary File 1, Supplementary File 2, Supplementary File 3).* -Were questions, prompts, guides provided by the authors? Was it pilot tested?

*Probes were included in the semi-structured data collection tool to solicit more in-depth responses. The use of probes is mentioned in the data collection paragraph, within the methods section. See Attached Supplementary File 1, Supplementary File 2, Supplementary File 3.*

*Due to the fact that data were collected in the height of COVID (December 2021-February 2022), the study team did not pilot the tools at sites, to avoid any additional exposure to COVID for the study team members and HCWs providing services. Instead, additional role plays were included in the data collection training.*

18. Repeat interviews -Were repeat interviews carried out? If yes, how many?

*Repeat interviews were not conducted.*

19. Audio/visual recording -Did the research use audio or visual recording to collect the data?

*Yes, in the data collection paragraph, within the methods section, we state that all interviews were audio-recorded.*

20. Field notes -Were field notes made during and/or after the interview or focus group?

*Yes, we have documented in the data collection paragraph, within the methods section, that field notes were written.*

21. Duration -What was the duration of the interviews or focus group?

*We have stated in the data collection paragraph, in the methods section, that interviews took between 50-90 minutes. (See Attached Supplementary File 1, Supplementary File 2, Supplementary File 3).*

22. Data saturation- Was data saturation discussed?

*In the data collection paragraph, within the methods section, we clarify that we followed recommendations from previous literature regarding the sample size needed to reach saturation (see citation below).*

*Guest, G., Bunce, A., & Johnson, L. (2006). How many interviews are enough? An experiment with data saturation and variability. Field Methods, 18(1), 59-82.* [*https://doi.org/10.1177/1525822X05279903*](https://doi.org/10.1177/1525822X05279903)

23. Transcripts returned -Were transcripts returned to participants for comment and/or correction?

*No. The study did not permit for contact with study participants after data collection*.

**Domain 3: analysis and findings**

***Data analysis***

24. Number of data coders -How many data coders coded the data?

*Two research assistants were analyzed the data under the supervision of the qualitative lead co-investigator. See analysis paragraph in methods section.*

25. Description of the coding tree -Did authors provide a description of the coding tree?

*The process of analysis is described in the analysis paragraph of the methods section. This was a short-answer analysis, which did not use a coding tree.*

26. Derivation of themes -Were themes identified in advance or derived from the data?

*Themes were derived from the data. See analysis paragraph in methods section.*

27. Software -What software, if applicable, was used to manage the data?

*No software was used. We have added language to the analysis section to state that the analysis was done manually. See analysis paragraph in methods section.*

28. Participant checking -Did participants provide feedback on the findings?

*No. The study did not permit for contact with study participants after data collection.*

***Reporting***

29. Quotations presented -Were participant quotations presented to illustrate the themes / findings? Was each quotation identified? e.g. participant number

*Yes, participate quotes were used. Instead of using a participant number, we included four components of identifying data to ensure readers that we are not citing the same participant multiple times, and provide more insight into who the participant was. This information is found following each of the quotes in the results section.*

30. Data and findings consistent- Was there consistency between the data presented and the findings?

*Yes. We have used quotes from participants to further illustrate this. See tables within results sections.*

31. Clarity of major themes -Were major themes clearly presented in the findings?

*Yes, it is clearly stated in the second paragraph of the results section that there are four major themes shared.*

32. Clarity of minor themes- Is there a description of diverse cases or discussion of minor themes?

*Within each of the four main themes, minor themes are discussed and represented with quotes in the tables following each of the themes. This is in the results section.*
