# Supplementary material for: Understanding health systems challenges in providing Advanced HIV Disease (AHD) care in a hub and spoke model: a qualitative analysis to improve AHD care program in Malawi
Source: BMC Health Serv Res. 2024 Feb 26;24:244. doi: 10.1186/s12913-024-10700-1 (PMC10897989; doi:10.1186/s12913-024-10700-1)
Supplement: Supplementary file 2 — Supplementary Material 2 [file 12913_2024_10700_MOESM2_ESM.docx]

## **Supplementary File 1:** In-depth Interview with Health Care Workers

Participant ID number __ __ - __ - ___ ___ ___ Interviewer Name: ___________________________

Site Name: ____________________________ Interview date: __ __ / __ __ / __ __ __ __ (*dd-mm-yyyy*)

Start time: _____: ______ End time: _____: _____ Duration of interview: _____ mins/_____ hrs.

*INSTRUCTIONS:* *This* *interview should only be started once written informed consent has been obtained from the participant. Read all of the questions and all of the information that is in bold print aloud to the participant. Use the probes as needed to gather more information from the participant. Probes should be used after the participant has spoken freely.*

**Thank you again for agreeing to participate in our study.**

**Demographic Information**

A1. Gender

Male  (1)

Female  (2)

A2. Which department do you work in?

ART clinic  (1)

Female ward  (2)

Male ward  (3)

TB ward  (4)

Other (*fill in*): __________________  (5)

A3. What is your position?

Nurse technician  (1)

Nurse Midwife Tech...  (2)

Registered Nurse  (3)

Registered Midwife  (4)

Reg Nurse Midwife  (5)

Laboratory Technician  (6)

Laboratory Technologist  (7)

Clinicians (MA/ CO/ MD)  (8)

Other (specify): __________________  (9)

A4. For how long have you been in this position at this facility or any other facility?

____Years…………… (For those less than one year, enter 0.)

A5. Is the facility you work in:  (1) A hospital  (2) A health center

**Facility capacity for advanced HIV patients**

1. How well-equipped is this facility to handle advanced HIV (AHD) patients?
   *Probe: Availability of medication, sufficient space at the facility to see patients, waiting space for patients, adequate supplies to take samples, adequate machines to test samples, etc.*
2. Does this facility have PoC machines on site?

2a) If yes, are the number of HCWs trained to use the PoC machine adequate?
 *Probe: Please explain why or why not.*

**Training**

1. Please describe the training you received on providing advanced HIV care.
   *Probe: What material was covered? Who conducted the training? How many days was the training? More than one training?*
2. How well did you feel that the training prepared you to screen and diagnose patients with advanced HIV?
   *Probe: Training was sufficient or not? Why or why not? What remaining questions did you have? Please describe anything that was unclear.*
3. How well did you feel that the training prepared you to diagnose patients with tuberculosis?
   *Probe: Training was sufficient or not? Why or why not? What remaining questions did you have?*
4. How well did you feel that the training prepared you to diagnose patients with cryptococcal meningitis?
   *Probe: Training was sufficient or not? Why or why not? What remaining questions did you have?*

**Patients and advanced HIV disease**

1. For the patients that were newly diagnosed as HIV-positive and as having advanced HIV, what are the reasons they provide for why they sought HIV testing and treatment so late?
   *Probe: Please describe any social factors such as stigma, lack of disclosure, fear, etc. Please describe any other barriers such as lack of time, concerns about cost, concerns about travel to the facility, etc.*

*7a) Do you think there are any differences among the challenges experienced among men vs. women? If yes, please describe.*

1. For the patients that were previously diagnosed with HIV, and who developed advanced HIV, what were their treatment challenges that resulted in them developing AHD?
   *Probe: Adherence challenges, side effects from the medication, confusion about the medication, challenges accepting their HIV status, challenges with disclosure, etc.*

8a) *Do you think there are any differences among the challenges experienced among men vs. women? If yes, please describe.*

1. How have patients responded when they were told that they had low CD4 count?
   *Probe: Accepting? Frustrated? Surprised/not surprised? Were there any differences among how men vs. women reacted to learning their AHD status?*
2. How have patients responded when they were told that they had opportunistic infection?
   *Probe: Accepting? Frustrated? Surprised/not surprised? Were there any differences among how men vs. women reacted to learning their AHD status?*
3. What are the challenges that patients face with opportunistic infections?
   *Probe: What kind of opportunistic infections do they get? What challenges do they experience treating these opportunistic infections?*
4. What are the most common challenges for AHD patients?
   *Probe: Treatment failure, side effects from the medications, challenges to travel to the facility so frequently, etc.*
5. How well do patients adhere to HCW recommendations regarding new medications and increased number of clinical appointments?
   *Probe: Are patients likely to abide by the recommendations? What influences this decision?*

13a. Please describe any differences between how male and female patients adhere to HCW recommendations.

**Site Level Questions**

**Spoke Sites:**

1. How comfortable are you receiving patients transferred to your site?
   *Probe: What were some of the challenges with these transfer patients?*
2. How comfortable are you supporting stable patients with advanced HIV disease?
   *Probe: Are there certain areas you are more comfortable with than other?*
3. How comfortable are you to transfer your unstable patients with advanced HIV to the Hub sites?
   *Probe: Did the patients have any concerns about the transfer? Did you have any concerns about the transfer?*
4. Please tell me, how does transferring your unstable advanced HIV patients affect your work?
   *Probe: Does transferring the unstable HIV patients create more/less work for you? How does this affect the amount of time you spend with your other patients?*

**Hub Sites:**

1. How comfortable are you supporting unstable patients with advanced HIV disease?
   *Probe: Are there certain areas you are more comfortable with than other?*
2. How comfortable are you receiving the transfers of the unstable patients with advanced HIV?
   *Probe: What are some of the challenges with these transfer patients?*
3. How does the volume of unstable patients affect your workload?
   *Probe: Do you feel that you had adequate time to care for these patients? Please describe the situation.*

**Treatment at Hub Sites**

1. How comfortable are you diagnosing cryptococcal meningitis among the AHD patients?
   *Probe: Are there any areas that are challenging? Taking samples, processing samples, etc.*
2. How well did you feel that the training prepared you to diagnose treatment failure and switch patients to second-third line of ART?
   *Probe: Were you comfortable providing the additional adherence counseling required? Please describe any challenges or additional information needed.*
3. How comfortable are you supporting patients with treatment failure?
   *Probe: Please tell us about any challenges addressing treatment failure (drug stocks-outs, toxicity, etc.)*

**Health care worker experience**

1. How comfortable are you with diagnosing tuberculosis among the AHD patients?
   *Probe: Are there any areas that are challenging? Taking samples, processing samples, etc.*
2. How well does the referral system to transfer AHD patients work?
   *Probe: What works well? Please describe any challenges.*
3. How has the improved AHD training/ services now provided at the facility changed your work load?

*Probe: Please describe any changes in your role, additional work/ decreased work, changes to the hours, etc.*

1. Has the new AHD register had any impact on your work?
   *Probe: How easy is it to use the register? Has it created more/less work?*
2. A quality improvement (QI) process has been created to share results of the AHD work at this facility in real time. Did this new system add any additional value? Please describe.
3. How has the improvements with AHD improved your satisfaction with your job?
   *Probe: Do you feel more empowered to care for sicker patients*?
4. Have you and HCWs at this facility received the necessary mentoring and support to provide AHD services?
   *Probe: If yes, please describe what mentoring support you’ve received. If no, please describe what support is needed.*
5. What challenges do you experience supporting AHD patients?
   *Probe: Need to initiate patients on new ART regimen in shorter periods of time, additional counseling required, additional appointments, etc.*
6. How do you overcome these challenges?

**Recommendations**

1. What can be done to improve your ability to provide care to AHD patients?
   *Probe: Please consider both facility infrastructure and your training.*
2. What can be done at the facility level to improve AHD care for patients?

*Probe: Please describe any additional services needed, supplies, infrastructure, etc.*

**Costing Questions**

1. How much time do you spend with a typical, **non-AHD** HIV patient? ___: ___ (minutes: hours)
2. How much time do you spend with a **stable** **AHD** patient? ___: ___ (minutes: hours)
3. How much time do you spend with an **unstable** **AHD** patient? ___: ___ (minutes: hours)

**Thank the participant for their time.**

Record end time at the top of the transcript.
