# Supplementary material for: Understanding health systems challenges in providing Advanced HIV Disease (AHD) care in a hub and spoke model: a qualitative analysis to improve AHD care program in Malawi
Source: BMC Health Serv Res. 2024 Feb 26;24:244. doi: 10.1186/s12913-024-10700-1 (PMC10897989; doi:10.1186/s12913-024-10700-1)
Supplement: Supplementary file 3 — Supplementary Material 3 [file 12913_2024_10700_MOESM3_ESM.docx]

## **Supplementary Material 1:** In-depth Interview with Advanced HIV Disease Patients

Participant ID number __ __ - __ - ___ ___ ___ Interviewer Name: ___________________________

Site Name: ____________________________ Interview date: __ __ / __ __ / __ __ __ __ (*dd-mm-yyyy*)

Start time: _____: ______ End time: _____: _____ Duration of interview: _____ mins/_____ hrs.

*INSTRUCTIONS:* *This* *interview should only be started once written informed consent has been obtained from the participant. Read all of the questions and all of the information that is in bold print aloud to the participant. Use the probes as needed to gather more information from the participant. Probes should be used after the participant has spoken freely.*

**Thank you again for agreeing to participate in our study.**

**Demographic Information**

A1. Gender

Male  (1)

Female  (2)

A2. Date of Birth______________ (If not known, Age: __ __ (years) (age at last birthday)

A3. What is your marital status?

Married /Living with partner  (1)

Never married / single  (2)

Separated/ Divorced/ Widow  (3)

A4. What is your level of education?

No school  (1)

Some primary  (2)

Completed primary  (3)

Some secondary  (4)

Completed secondary  (5)

Some tertiary  (6)

Completed tertiary  (7)

A5. Have you just discovered your HIV positive status in the last few visits to the health facility?

Yes  No

A6. If no, for how many years have you known your HIV-positive status? ______ (years). For those less than one year, enter 0.

A7. If you are on ART, for how many years have you been on ART? ______ (years). For those less than one year, enter 0.

A8. If on ART, have you changed the medications you are taking for ART?

Yes  (0)

No  (1)

A9. Were you aware that your CD4 count was low or that you have a high viral load before your last appointment at the HIV clinic?

Yes  (0)

No  (1)

A10. Are you aware of any opportunistic infections you may have?

No  (0)

Yes  (1)

A11. If yes, which opportunistic infections? (Select all that apply)

Tuberculosis  (1)

Cryptococcal Meningitis  (2)

Kaposi sarcoma  (3)

Other  (4) (Please specify: ______________________________)

**Questions for NEWLY diagnosed HIV patients**

1. Before learning your HIV status, did you suspect that you may be HIV-positive?
   *Probe: Please tell me why you felt this way. If participant suspected an HIV-positive status, ask what delayed them in coming for a HIV test.*
2. What encouraged you to test for HIV now?
   *Probe: Please describe any considerations such as your health condition, family/partner influence, health facility considerations, etc.*
3. How did you respond when you initially learned your HIV status?

*Probe: Were you surprised or suspecting a positive-status? Did you easily accept or struggle to accept your HIV status?*

1. Have you disclosed your HIV status to your partner or family?
   *Probe: Why or why not? Do you plan to?*
2. HIV diagnoses are called ‘advanced HIV’ when the disease has progressed, the CD4 count is low, the viral load is high and immunity is decreased. Were you surprised to learn that your HIV is advanced?
   *Probe: Why or why not?*

**Questions for PREVIOUSLY diagnosed HIV patients**

1. How did you respond when you initially learned your HIV status?

*Probe: Were you surprised or suspecting a positive-status? Did you easily accept or struggle to accept your HIV status?*

1. Did you disclose your HIV status to your partner or family?
   *Probe: Why or why not? Do you plan to?*
2. Please think back to when you were first told about antiretroviral treatment (ART). What was your response to learning that you needed to start treatment?
   *Probe: Were you accepting of treatment? Reluctant to start treatment? Please tell me why you felt this way.*
3. Please tell me about your experience taking ART, once you started ART.
   *Probe: How was your experience on ART? (Easy/challenging). Did you understand the guidance from the HCWs?*
4. Did you have interruptions in taking your ART?
   *Probe: How many times have you started and stopped treatment again?*
   1. *When there was an interruption in treatment, on average how long did the interruption last?*
   2. What were the main reasons for your interruption(s)?

*Probe: Please describe any concerns about stigma, the health facility, cost of travel to the facility, problems with the medications, etc.*

- 1. What encouraged you to return to care?
     *Probe: Please describe any considerations such as your health condition, family/partner influence, health facility considerations, etc.*

1. HIV diagnoses are called ‘advanced HIV’ when the HIV disease has progressed, meaning the CD4 count is low, the viral load is high and immunity is decreased. Before you learned that you had advanced HIV, what you heard causes one’s HIV infection to become advanced HIV?

*Probe: Did you know what makes HIV become advanced? Can you list the causes you know?*

1. Were you aware that this a need to monitor your immune status?
   *Probe: Please tell me what you have heard about how to monitor your immune status.*
2. What had you heard about decreased immunity because of HIV and the risk of opportunistic infections?
   *Probe: Did you know that HIV can weaken your immune response and put you at risk to get certain infections and diseases?*
3. Did you know that there are medications to prevent some of the infections that can happen when you have advanced HIV?
   *Probe: If yes, what infections have you heard can be prevented?*
4. Were you surprised to learn that your HIV is advanced?
   *Probe: Why or why not?*
5. What actions did you take to prevent developing advanced HIV?
   *Probe: Taking medication on schedule, refilling medication on time, following dosing instructions, etc.*
6. How have your medications changed with being diagnosed with advanced HIV?
   *Probe: What additional medications will you take? How will your current medications change?*
7. How have your visits to the health facility changed with being diagnosed with advanced HIV?
   *Probe: Will you come more/less often? Will you visit different units?*

**Guidance from health care workers about advanced HIV**

1. What did HCWs explain about the challenges of advanced HIV?
   *Probe: Opportunistic infections (tuberculosis, cryptococcal meningitis, preventive treatment for opportunistic infections, switch of ART and enhanced counseling support)*
2. How do you feel about having advanced HIV?
   *Probe: Okay, neutral, appreciative for recognizing the problem, eager to start treatment, worried, stressed, etc.*
3. What could help you to feel better about your advanced HIV status?
   *Probe: additional information, speaking with others with advanced HIV, changing the drug regimen, etc.*
4. What additional information would you have liked to have received from the HCWs?

*Probe: Information on what ‘is’ advanced HIV, treatment, side effects, clinic appointments, questions about the ability to live with advanced HIV, etc.*

1. Please tell me how involved the HCWs were with helping you make decisions about your health care.
   *Probe: Did you feel like you had the ability to make decisions about your own care?* *Please describe.*
2. Did you trust the advice of the HCWs?
   *Probe: If yes, what encouraged you to trust the HCWs? If no, what caused you to not trust the HCWs?*

**Living with advanced HIV**

1. What do you think might be some challenges to following the HCW’s advice?
   *Probe: Taking additional medications, attending additional clinic appointments, challenges at the facility (such as stock-outs, lack of services), etc.*
2. How will you overcome some of these challenges?
   *Probe: Creating ‘reminders’ to take medication, overcoming challenges attending additional clinic appointments, increasing family support, etc.*
3. What additional support would you like to receive at the health facility?
   *Probe: Additional counseling, additional support groups, support of lay staff, etc.*

**Recommendations**

1. How could the care at this facility be improved to strengthen patients experience with advanced HIV?
   *Probe: Improve wait times, waiting space, care provided by HCWs, ART pick-up, counseling, etc.*

**Costing Questions**

1. When were you first diagnosed with advanced HIV ? __________________ (list month and year)

1. How many trips have you made to the facility since being diagnosed with Advanced HIV Disease (AHD)? ____________ (number of trips)
2. How long did your most recent visit to the health facility take ? Please think about the time that passed from when you left home to when you returned (total time) ? _____ minutes : _____ hours
3. What was the cost of transport (including return) for your most recent visit to the health facility? ______________ (Give monetary amount in MWK)
4. Were you accompanied by another person when you came to the facility ? ____ Yes ____ No
5. Besides transport, what out-of-pocket costs were you responsible for, if any, in order to attend your last medical outpatient visit ? (Give monetary amount, and check any item that applies, and / or insert a comment with more explanation).

List can include (tick box to left, and enter amount to the right

- - - Gloves ________________________
    - Bedpan ________________________
    - Fee for radiology________________
    - Fee for tests ____________________
    - Fee for other procedures __________
    - Fee for medicines ________________
    - Other fees ? _____________________ (list item and cost)

4 Can you estimate how much these out-of-pocket costs add up to, in the past 3 months ? ___________(Answer in MWK)

5 When traveling to the facility in this past quarter, was cost a challenge ? (Yes/ No)

6 a Have you lost income as a result of your HIV diagnosis ? (Yes/ No)

If yes, please explain why : ____________________________________________________________

6 b If Yes to above, can you estimate the amount of this lost income over a three month period ? _______________ (Answer in MWK)

6 c What is your annual household income. This amount is referring to how much money is everyone in your family that is working earns combined for a one year period. ___________ (Answer in MWK)
