# Supplementary material for: Understanding health systems challenges in providing Advanced HIV Disease (AHD) care in a hub and spoke model: a qualitative analysis to improve AHD care program in Malawi
Source: BMC Health Serv Res. 2024 Feb 26;24:244. doi: 10.1186/s12913-024-10700-1 (PMC10897989; doi:10.1186/s12913-024-10700-1)
Supplement: Supplementary file 4 — Supplementary Material 4 [file 12913_2024_10700_MOESM4_ESM.docx]

## **Supplementary File 3:** In-depth Interview with Lay Cadre

Participant ID number __ __ - __ - ___ ___ ___ Interviewer Name: ___________________________

Site Name: ____________________________ Interview date: __ __ / __ __ / __ __ __ __ (*dd-mm-yyyy*)

Start time: _____: ______ End time: _____: _____ Duration of interview: _____ mins/_____ hrs.

***INSTRUCTIONS****:* *This* *interview should only be started once written informed consent has been obtained from the participant. Read all of the questions and all of the information that is in bold print aloud to the participant. Use the probes as needed to gather more information from the participant. Probes should be used after the participant has spoken freely.*

**Thank you again for agreeing to participate in our study.**

**Demographic Information**

A1. Gender

Male  (1)

Female  (2)

A2. What is your age? __________________

A3. Which type of lay staff are you?

Expert Client (EC)  (1)

HIV Diagnostic Assistant (HDA)  (2)

HIV Surveillance Assistant (HSD)  (3)

Other (*fill in*): __________________  (4)

A4. For how long have you been in this position at this facility or any other facility?

____Years…………… (For those less than one year, enter 0.)

A5. Is the facility you work in:  (1) A hospital  (2) A health center

**Training & Role**

1. Please describe the training you received on providing advanced HIV care (AHD).
   *Probe: What material was covered? Who conducted the training? How many days was the training? More than one training?*
2. Please describe your role at this facility regarding care of AHD patients.

*Probe: Please describe all activities you do including counseling, home visits, etc.*

1. Have you and other lay staff at this facility received the necessary mentoring and support to provide AHD services?
   *Probe: If yes, please describe what mentoring support you’ve received. If no, please describe what support is needed.*

**Patients and advanced HIV disease**

1. For the patients that were newly diagnosed as HIV-positive and as having advanced HIV, what are the reasons they provide for why they sought HIV testing and treatment so late?
   *Probe: Please describe any social factors such as stigma, lack of disclosure, fear, etc. Please describe any other barriers such as lack of time, concerns about cost, concerns about travel to the facility, etc.*

*6a) Do you think there are any differences among the challenges experienced among men vs. women? If yes, please describe.*

1. For the patients that were previously diagnosed with HIV, and who developed advanced HIV, what were their adherence challenges that resulted in them developing AHD?
   *Probe: Side effects from the medication, confusion about the medication, challenges accepting their HIV status, challenges with disclosure, etc.*

7a) *Do you think there are any differences among the challenges experienced among men vs. women? If yes, please describe.*

1. How well do patients adhere to HCW recommendations regarding new medications and increased number of clinical appointments?
   *Probe: Are patients likely to abide by the recommendations? What influences this decision?*

*6a. Do you think there are any differences among likelihood to follow the HCWs recommendations between men and women? Please explain.*

1. What are the most common challenges for AHD patients?
   *Probe: Treatment failure, side effects from the medications, challenges to travel to the facility so frequently, etc.* *Are there any differences between the challenges that the male and female patients experience?*

**Patient Transfer Questions**

**Spoke Sites:**

1. How comfortable are you receiving patients transferred to your site?
   *Probe: What were some of the challenges with these transfer patients?*
2. How comfortable are you supporting stable patients with advanced HIV disease?
   *Probe: Are there certain areas you are more comfortable with than other?*
3. How comfortable are you to transfer your unstable patients with advanced HIV to the Hub sites?
   *Probe: Did the patients have any concerns about the transfer? Did you have any concerns about the transfer?*

**Hub Sites:**

1. How comfortable are you supporting unstable patients with advanced HIV disease?
   *Probe: Are there certain areas you are more comfortable with than other?*
2. How comfortable are you receiving the transfers of the unstable patients with advanced HIV?
   *Probe: What were some of the challenges with these transfer patients?*
3. How does the volume of unstable patients affect your workload?
   *Probe: Does having many unstable patients significantly increase your workload? Please describe the situation.*

**Lay cadre experience**

1. How well does the referral system to transfer AHD patients work?
   *Probe: What works well? Please describe any challenges.*
2. A quality improvement (QI) process has been created to share results of the AHD work at this facility in real time. Did this new system add any additional value? Please describe.
3. How has the improvement of the AHD program improved your satisfaction with your job?
   *Probe: Do you feel more empowered to care for sicker patients*?
4. When you are counseling the patients, what are topics/areas that they require more information?

*Probe: What topics are the least clear to them and need reinforcement?*

19a) *Do you think there are any differences among the challenges experienced among men vs. women? If yes, please describe.*

1. Please tell me about any strategies you have used to support the counseling of AHD patients

*Probe: Please speak freely about any techniques or tips you or other counselors have developed or recommended to patients.*

1. What challenges do you experience supporting AHD patients?
   *Probe: Please include any challenges, such as not knowledgeable enough about certain topics, lack of adequate space, time, materials to share with the participants, enough lay staff to support all of the patient counseling needs, etc.*
2. How do you overcome these challenges?

**Recommendations**

1. How can the counseling be improved at the facility to strengthen patients’ adherence?
   *Probe: Please consider any materials, additional time needed, additional home visits, changing the counseling structure, etc.*
2. What can be done to improve your ability to provide care to AHD patients?
   *Probe: Please consider both facility infrastructure and your training.*
3. What can be done at the facility level to improve AHD care for patients?

*Probe: Please describe any additional services needed, supplies, infrastructure, etc.*

**Thank the participant for their time.**

Record end time at the top of the transcript.
